# Efficient computation of spaced seed hashing with block indexing

**DOI:** 10.1186/s12859-018-2415-8

**Published:** 2018-11-30

**Authors:** Samuele Girotto, Matteo Comin, Cinzia Pizzi

**Affiliations:** Department of Information Engineering, University of Padova, via Gradenigo 6/A, Padova, Italy

**Keywords:** Spaced seeds, k-mers, Efficient computation of hashing

## Abstract

**Background:**

Spaced-seeds, i.e. patterns in which some fixed positions are allowed to be wild-cards, play a crucial role in several bioinformatics applications involving substrings counting and indexing, by often providing better sensitivity with respect to *k*-mers based approaches. K-mers based approaches are usually fast, being based on efficient hashing and indexing that exploits the large overlap between consecutive *k*-mers. Spaced-seeds hashing is not as straightforward, and it is usually computed from scratch for each position in the input sequence. Recently, the FSH (Fast Spaced seed Hashing) approach was proposed to improve the time required for computation of the spaced seed hashing of DNA sequences with a speed-up of about 1.5 with respect to standard hashing computation.

**Results:**

In this work we propose a novel algorithm, Fast Indexing for Spaced seed Hashing (FISH), based on the indexing of small blocks that can be combined to obtain the hashing of spaced-seeds of any length. The method exploits the fast computation of the hashing of runs of consecutive 1 in the spaced seeds, that basically correspond to *k*-mer of the length of the run.

**Conclusions:**

We run several experiments, on NGS data from simulated and synthetic metagenomic experiments, to assess the time required for the computation of the hashing for each position in each read with respect to several spaced seeds. In our experiments, FISH can compute the hashing values of spaced seeds with a speedup, with respect to the traditional approach, between 1.9x to 6.03x, depending on the structure of the spaced seeds.

**Electronic supplementary material:**

The online version of this article (10.1186/s12859-018-2415-8) contains supplementary material, which is available to authorized users.

## Background

*k*-mers counting, indexing and searching are fundamental operations at the very basis of many bioinformatics tools. A most notable example is their exploitation on sequence similarity search for which the “hit-and-extend” method introduced by BLAST [1] led to a revolutionary fast and sensitive approach for local alignment. In the “hit” step exact matches of *k*-mers (*k*=11 for DNA) between two sequences are detected. Next, potential candidates are extended to obtain a local alignment with high statistical significance. BLAST has long been one of the most used tools for the analysis of omics sequences.

*k*-mers profiles are also widely used in alignment-free techniques [2] for the definition of statistical scores for sequence comparison [3, 4], finding application on a broad range of bioinformatics problems (e.g. [5–13]), and pushing the development and usage of time and space efficient algorithms and data structures for *k*-mer counting and indexing (e.g. [14–18]).

Although the matching of contiguous *k*-mers is largely used in sequence analysis, the use of not consecutive matches, i.e. *spaced seeds*, can lead in principle to more sensitive results [19]. This is because spaced seeds offer the advantage, with respect to *k*-mers, of considering positions that are not consecutive, hence statistically less dependent. On the other side, the problem of maximizing the spaced seeds sensitivity is known to be NP-hard [20]. The design of effective spaced seeds has been addressed in several studies [21–24]. Nowadays, spaced seeds have replaced traditional *k*-mers based approaches in the design of state-of-the-art solutions to several problems that involve sequence comparison. Among others we can enlist: phylogenetic tree reconstruction [25], protein classification [26], mapping of reads [27], multiple sequence alignment [28], metagenomics binning and classification [29–31]. The literature on spaced seeds is vast, and we refer the interest reader to [32] for a survey.

Several routine operations on large scale sequence analysis, including building and querying indexes, and searching for similarity among sequences, are based on *k*-mers counting. In order to speed-up *k*-mers counting, hashing is often used. In fact, hashing consecutive *k*-mers is fast and simple, since the hash of a *k*-mer starting at position *i* can be computed from the hash of the *k*-mer at position *i*−1 with few operations, since they share *k*−1 symbols [33].

Unfortunately, this property no longer holds for spaced seeds, due to the presence of “don’t care” positions, leading to a slowdown of the whole analysis. A good example of this effect is the metagenomic read classifier Clark [10]. Its spaced seed counterpart, Clark-S [31], has a better classification quality, but a drop from 3.5M to 200k reads per minute on classification rate with respect to Clark. Slow downs when using spaced seeds has also been shown in [26, 27, 29].

The problem of speeding up the computation of spaced seed hashing for each position in a given sequence was recently addressed in [34, 35] where FSH, an approach based on spaced seed self-correlation, was proposed reporting a speed-up of 1.5x, on average, with respect to the standard way to compute spaced seed hashing. In this paper we address the same problem, considering the Rabin-Karp rolling hash.

The novel approach we present here, FISH, is based on the decomposition of the spaced seed mask into blocks of consecutive 1s. These blocks represent contiguous matches, i.e. *k*-mers of the specified length. Since the hashing of *k*-mers is a very fast operation, we reduced the problem of spaced seed hashing to the problem of hashing its *k*-mer components and then combined them in order to obtain the hashing of the complete spaced seed. We performed a wide set of experiments, using several spaced seeds, varying in terms of length and weight, and NGS datasets with different read lengths. Our approach proved to be faster than the standard approach, and also of FSH. We extended our algorithm and experiments also to the multiple spaced seed hashing framework, obtaining an average speed-up with respect to standard indexing of 6x.

In the next sections we will present our approach and the results of our experiments, discussing the performances of our approach under different settings.

## Methods

In this section we start by recalling some formal definitions about spaced seeds through the notation introduced in [36], and then we will describe our algorithm to compute the spaced seed hashing of each position in a given input string, a fundamental step in many applications [25–29, 31].

### Fundamental concepts on spaced seeds

#### **Definition 1**

(Spaced seed.) A *spaced-seed S* (or just a seed) is a binary string of length *k*, where the symbol ‘*1*’ requires a match in that position, while a symbol ‘*0*’ allows for “don’t care”. A spaced seed is characterized by its length *k* and by its weight *W*<*k*, which is the number of 1s in the string. A spaced seed always begins and ends with a *1*.

#### **Definition 2**

(The shape Q of a spaced seed.) The *shape Q* of a spaced seed is the set of non negative integers that correspond to the positions of the spaced seed where there is a *1*. The shape *Q* can describe a spaced seed completely: the weight *W* is equal to |*Q*|, and its span (or length) *s*(*Q*) is given by max*Q*+1.

#### **Definition 3**

(The positioned shape *i*+*Q*.) Given any integer *i* and shape *Q*, we define the positioned shape *i*+*Q* as the set {*i*+*k*,*k*∈*Q*}.

#### **Definition 4**

(*Q*-gram.) For any position *i* in the string *x*=*x*_0_*x*_1_…*x*_*n*−1_, with 0≤*i*≤*n*−*s*(*Q*), let us consider the positioned shape *i*+*Q*={*i*_0_,*i*_1_,…,*i*_*W*−1_}, where *i*_0_<*i*_1_<...<*i*_*W*−1_. The *Q*-gram *x*[*i*+*Q*], starting at position *i* in *x*, is the string of length |*Q*| described by $x_{i_{0}} x_{i_{1}} \dots x_{i_{W-1}}$.

**Example** Let us consider the string *x*=*ACTGACTGGATTGAC*, and a spaced seed 1101110011111. Then the shape of the spaced seed is *Q*={0,1,3,4,5,8,9,10,11,12}, its weight is |*Q*|=10 and its span is *s*(*Q*)=13. The *Q*-gram *x*[0+*Q*] is given by the concatenation of the symbols that occur at positions 0+*Q*={0,1,3,4,5,8,9,10,11,12}, *x*[ 0+*Q*]=*ACGACGATTG*:


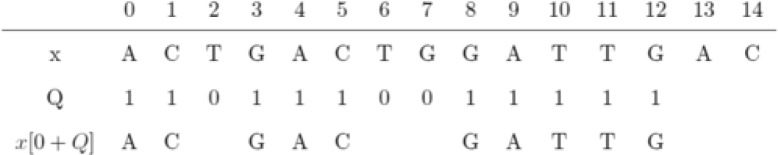


Similarly the other *Q*-grams are given by the concatenations of the symbols at positions 1+*Q*={1,2,4,5,6,9,10,11,12,13}: *x*[1+*Q*]=*CTACTATTGA*; and 2+*Q*={2,3,5,6,7,10,11,12,13,14}: *x*[2+*Q*]=*TGCTGTTGAC*.

Now we can formally state our problem such as:

#### **Problem 1**

Let *x*=*x*_0_*x*_1_…*x*_*i*_…*x*_*n*−1_ be a string of length *n*, *Q* be a spaced seed, and *h* be a hash function that maps a string into a binary codeword. Compute the hash $\mathcal {H}(x,Q)$ for each *Q*-gram of the string *x*, following in the natural order from the first position 0 to the last position *n*−*s*(*Q*).

$${}\mathcal{H}(x,Q) = \langle h(x[0+Q]), h(x[1+Q]), \dots h(x[n-s(Q)]) \rangle $$

### Spaced seed hashing

The first step when computing the hash of a string defined over an alphabet $\mathcal {A}$ is to encode it into a binary string. For genomic sequences the simplest encoding consists in the definition of a function *encode* which maps the four nucleotides as follows: *encode*(*A*)=00,*encode*(*C*)=01,*encode*(*G*)=10,*encode*(*T*)=11. Given this mapping, we can compute the encodings of all symbols of the *Q*-gram *x*[0+*Q*]:

$$\begin{array}{ccccccccccc} x[0+Q] &A&C&G&A&C&G&A&T&T&G\\ {encodings}&00&01&10&00&01&10&00&11&11&10 \end{array} $$

Here we focus on the efficient computation of the Rabin-Karp rolling hash. In the case of DNA sequences since $|\mathcal {A}|=4$ is a power of 2, the multiplications can be implemented with a shift operation. More formally, for any given position *i* of the string *x*=*x*_0_*x*_1_…*x*_*n*−1_, we define the hashing *h*(*x*[ *i*+*Q*]) of the *Q*-gram *x*[*i*+*Q*] as:

1 $$ h(x[\!i+Q]) = \bigvee_{k \in Q} \left[(encode(x_{i+k}) \ll (m(k)*{log}_{2}|\mathcal{A}|)\right]  $$

where *m*(*k*)=|{*i*∈*Q*, such that*i*<*k*}|, i.e. given a position *k* in the spaced seed, *m*(*k*) holds the number of 1s to the left of *k*. Since each symbol is encoded with 2 bits, $m(k)*{log}_{2}|\mathcal {A}|$ gives the number of shifts to set the encoding of the *k*-th symbol in the right position.

In Table 1 we report a step-by-step computation of hashing value for the *Q*-gram *x*[0+*Q*] (up to length 6 just for page width limits constrains). With respect to the above example, the hashing value associated to the *Q*-gram *ACGACGATTG* simply corresponds to the list of encodings in Little-endian: 10111100100100100100. The hashing values for the others *Q*-grams can be determined through the function *h*(*x*[ *i*+*Q*]) with a similar procedure. Following the above example the hashing values for the *Q*-grams *x*[ 1+*Q*]=*CTACTATTGA* and *x*[ 2+*Q*]=*TGCTGTTGAC* are, respectively, 00101111001101001101 and 10001011111011011011.

**Table 1:** Step-by-step computation of the encoding of the prefix of length 6 of the *Q*-gram *x*[ 0+*Q*] in little-endian notation using Eq. (1)

	0	1	2	3	4	5	6	7	8	9
x	A	C	T	G	A	C	T	G	G	A
Q	1	1	0	1	1	1	0	0	1	1
m	0	1	2	2	3	4	4	5	5	6
Shifted-encodings	00	01 ≪ 2		10 ≪ 4	00 ≪ 6	01 ≪ 8			10 ≪ 10	
		0100								
				100100						
					00100100					
						0100100100				
									100100100100	

The Rabin-Karp rolling hash is very intuitive. However, other hashing functions, that can be more appropriate because they have some properties such as universality, uniform distribution in the output space, and higher-order independence [33], can be computed in a similar way. For example, one could use the cyclic polynomial rolling hash by replacing: shifts with rotations, OR with XOR, and the function *encode*(·) in Eq. (1) with a seed table where the letters of the DNA alphabet are assigned different random 64-bit integers.

Equation (1) can be directly used to address Problem 1 by applying it at each position in *x*. However, for each position the computation of the hashing function *h*(*x*[*i*+*Q*]) requires to extract and encode a number of symbols that is equal to the weight of the seed |*Q*| or, in other words, each symbol of *x* is read and encoded into the hash |*Q*| times. Therefore this solution can be very time consuming.

### Computing spaced seed hashing with block indexing

In the following we describe our contribution for the computation of hashing values through Fast Indexing of Spaced seeds Hashings (FISH). Let *Q*={*i*_1_,*i*_2_,…*i*_*k*_} be a spaced seed. It can be viewed as a series of runs of 1s, or unit blocks, interspersed with runs of 0s. First, we disassemble *Q* into its constituents unit blocks and we define the set *B* of starting positions of the unit blocks as:

$$B = \{0\} \cup \left\{i_{j} \in Q\setminus\{0\} \text{ such that } i_{j}-i_{j-1} >1\right\} $$

Given *B*={*b*_1_,*b*_2_,…,*b*_*t*_}, let *B*_*L*_={*l*_1_,*l*_2_,…,*l*_*t*_} be the (ordered) set of the lengths corresponding to each unit block. To compute the hashing of a spaced seed on a sequence *x* of length *n*, the FISH algorithm will scan *x* for fast hashing of *l*-mers whose lengths are in *B*_*L*_. For each length *l*∈*B*_*L*_ an array *T*_*l*_ of length *n*−*l*+1 is built where at position *i* the hash of the l-mer *x*[ *i*,*i*+*l*−1] is stored. This pre-processing is very fast, as it can exploit the large overlap (*l*−1 symbols) between consecutive *l*-mers in order to compute the hashing of consecutive positions in constant time.

Then, to compute the hash of the Q-gram identified by the position shape *i*+*Q*, we proceed as follows. For each unit block *b*_*j*_ of length *l*_*j*_ we look up at the array $T_{l_{j}}$, and specifically to the value stored at position *i*+*b*_*j*_. Let *h*_*j*_ be such value. The hashing of the Q-gram is then computed by shifting *h*_*j*_ of 2×*m*(*b*_*j*_) positions to the left. This process is repeated for all unit blocks and the contributions of each block are summed (bitwise OR).

#### **Example 1**

**Fig. 1:**
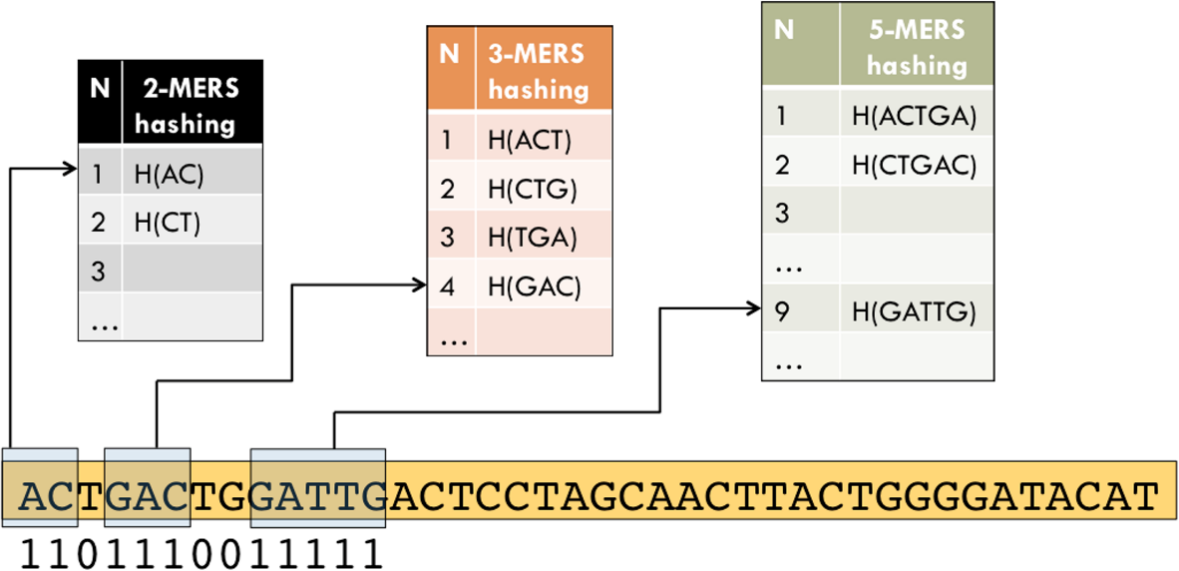
The hashing of each unit block in the spaced seed is looked up in the corresponding length *k*-mer table

Let us consider again the string *x*=*ACTGACTGGATTGACTCC* and the spaced seed *S*=1101 110011111, with associated shape *Q*={0,1,3,4,5,8,9,10,11,12},*m*={0,1,2,2,3,4,4,5,5,6,7,8,9,10}, and blocks with starting positions *B*={0,3,8}, and lengths *B*_*L*_={2,3,5}. To compute the hashing of the *Q*-gram *x*[0+*Q*] we must look up at *T*_2_[0] to retrieve the value of *h*_1_=0100, at *T*_3_[3] to retrieve the value of *h*_2_=010010, and at *T*_5_ to retrieve *h*_3_=1011110010 (see Fig. 1).

Then the hashings need to be combined to obtain the final hash value of *x*[ 0+*Q*]:

$$\begin{array}{lll} {}H(ACGACGATTG) &=& (h_{1} \! \ll \! 2 \cdot m(b_{1}))\! \vee \! (h_{2} \! \ll \! 2 \cdot m(b_{2})) \! \vee \! (h_{3} \! \ll \! 2 \! \cdot \! m(\!b_{3}\!))\\ &=& (0100 \ll 0) \vee (010010 \ll 4) \vee (1011110010 \ll 10)\\ &=& 10111100100100100100\\ \end{array} $$

### Computing multiple spaced seed hashing with block indexing

In some applications (for example [25, 29–31, 37]) using several spaced seeds increases the sensitivity of the results. In such a context, the FISH algorithm can be further exploited to improve the speed up with respect to the computation of the *Q*-grams hashing of each spaced seed separately. In fact, if two spaced seeds share a unit block of the same length *l*, we will need to compute the hashing of the *l*-mers of the input string just once, and then access the corresponding array *T*_*l*_ when computing the full hash of *Q*-grams for the two different spaced seeds.

More formally, let *Q*_1_,*Q*_2_,…*Q*_*n*_ be *n* spaced seeds. Let $B_{L}^{Q_{i}} =\{l_{1}^{Q_{i}}, l_{2}^{Q_{i}}, {,} \dots, l_{t_{i}}^{Q_{i}} \}$ be the set of lengths of the unit blocks of the spaced seed with shape *Q*_*i*_, for *i*=1,…,*n*. Let $\tilde {B}_{L} =\cup _{i=1}^{n} B_{L}^{Q_{i}}$ be the superset of all different unit block lengths among the spaced seeds we are considering. We will compute the hashing tables of each *l*-mer, with $l \in \tilde {B}_{L}$, in the input string *x* just once. These tables will be used for all spaced seeds so that if two spaced seeds share a unit block, the corresponding table will be computed only once. When we need to reconstruct the hash for the *Q*-gram intercepted by the spaced seed *Q*_*i*_ at position *j* in *x*, i.e. *x*[*j*+*Q*_*i*_], FISH will proceed as before by looking up at the *T*_*l*_ corresponding to the lengths of the blocks in the spaced seed *Q*_*i*_.

## Results

In this section we will discuss the time performance of the block indexing based approach FISH, presented here, and the FSH approach [35]. The speed ups are computed with respect to the time needed for the standard computation of spaced seeds hashing, where the hashing of each *k*-mer intercepted by the spaced seed is computed separately for each position in the input string as in Eq. (1).

### Spaced seeds and datasets description

In order to evaluate the performance of FISH we design a series of tests with different type of spaced seeds and various reads datasets. For our experiments we used the same spaced seeds and datasets used in [34] covering three types of spaced seeds: i) maximizing the hit probability [31]; ii) minimizing the overlap complexity [23]; and iii) maximizing the sensitivity [21].

In line with previous studies, we evaluate nine spaced seeds, three for each category. The spaced seeds used for this test are shown in Table 2. All spaced seeds *Q*1−*Q*9 (see Table 2) have the same weight |*Qi*|=22 and length *L*=31.

**Table 2:** The nine spaced seeds used in the experiments grouped according to their type

Spaced seeds maximizing the hit probability [31]	
Q1	1111011101110010111001011011111
Q2	1111101011100101101110011011111
Q3	1111101001110101101100111011111
Spaced seeds minimizing the overlap complexity [23]	
Q4	1111010111010011001110111110111
Q5	1110111011101111010010110011111
Q6	1111101001011100111110101101111
Spaced seeds maximizing the sensitivity [21]	
Q7	1111011110011010111110101011011
Q8	1110101011101100110100111111111
Q9	1111110101101011100111011001111

In order to evaluate FISH under different conditions, we build several sets of spaced seeds with rashbari, with different lengths from 16 to 45 and weights from 11 to 32. A complete list of spaced seeds is reported in the Additional file 1: Tables S1–S5.

As for the reads data to be scanned and hashed, we consider a series of datasets of metagenomic reads already used for classification and binning [9, 38]. We use synthetic metagenomic datasets (MiSeq, HiSeq, MK_a1, MK_a2, and simBA5) as well as simulated metagenomic datasets (S,L,R). The datasets (*R*_*x*_) simulate single-end long reads from Roche 454, with length 700 bp, and sequencing error of 1%. While the datasets (*S*_*x*_ and *L*_*x*_) are paired-end reads of short length (80 bp) following Illumina error profile. The synthetic metagenomic datasets are built from real shotgun reads of different species to mimic various microbiome communities. Furthermore, for the comparison of spaced seeds with different weights and lengths, we generated datasets of increasing read length of 100, 200, and 400 bp with Mason simulator [39] according to Illumina error profile. A summary of the datasets used in this study is reported in Table 3. All methods have been tested on a laptop with 16 GB RAM and Intel i74510U cpu at 2GHz.

**Table 3:** Number of reads and average lengths for each of the dataset used in our experiments

Datasets	Number of reads	Avg. read length
S6	1426457	80
S7	3307100	80
S9	4468336	80
S10	9981172	80
L5	1016418	80
L6	1182178	80
HiSeq	9989713	91
simBA5	5439738	100
MixK1	9629886	101
MixK2	7149900	101
MiSeq	9933556	131
R7	290473	702
R8	374576	715
R9	588256	715

### Analysis of speed up

**Fig. 2:**
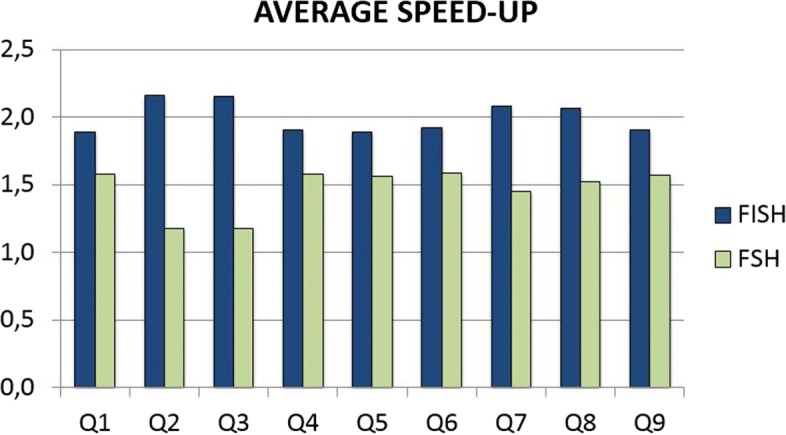
The speedup of FISH and FSH with respect to the standard hashing computation, as a function of the spaced seeds used in our experiments

In the first test we compare the performance of FISH with FSH in terms of speed up with respect to the standard hashing computation. In Fig. 2 we report the average speed ups on all datasets, for each spaced seed, obtainable with FISH and FSH approaches.

We can observe that FISH is faster than FSH independently on the spaced seed considered. As a reference, the standard approach (Eq. (1)), requires about 17 minutes to perform the hashing of a seed on all datasets. The two methods FISH and FSH can compute the hashings in 8.5 and 12 minutes respectively, with a speed up of 2 (FISH) and 1.46 (FSH). We noticed that the speed up can vary between spaced seeds, in fact FSH obtains speed ups in the range [1.18-1.58] and FISH in the interval [1.89-2.16]. As expected, the speed up depends on the structure of spaced seed to be hashed, however FSH seems to be highly dependent on the structure with a variation of 0.4 between minimum and maximum speed up, instead FISH variation is only 0.27. In summary, in this first experiments FISH in not only faster, but also less dependent of the spaced seed.

**Fig. 3:**
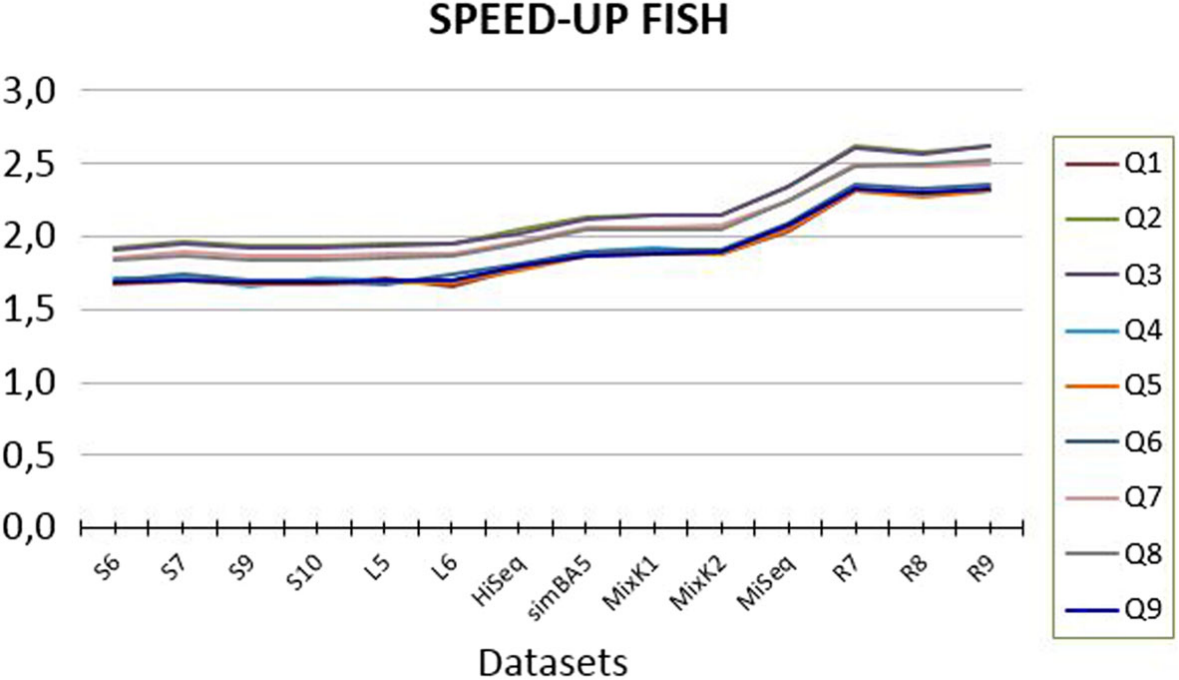
Details of the speedup of FISH on each of the considered datasets, ordered by reads length

To have a better understanding of the behavior of FISH on all datasets, Fig. 3 reports the performance of FISH for each datasets.

We noticed that the seeds with the best performance are Q2 and Q3, the top two lines in Fig. 3. However, all spaced seeds show a similar behavior across different datasets. The maximum difference between the best seed, top line, and the worse seed, bottom line, remains constant for each datasets confirming the robustness of FISH. Another interesting observation is that the speed up tends to increase with the reads length and it reaches the maximum performance on the long read (see R7, R8 and R9). A possible reason for this behavior is that these datasets contain long reads, and the impact of the initial transient is reduced.

**Fig. 4:**
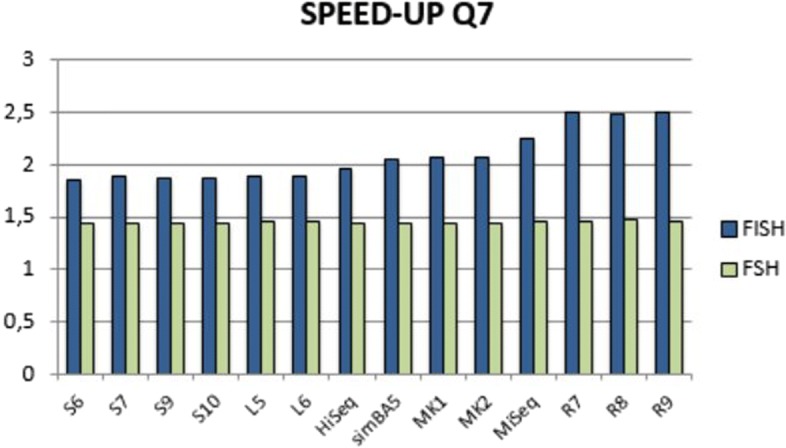
Details of the speedup of FISH and FSH on the spaced seed Q7 for each of the considered datasets, ordered by reads length

In Fig. 4 we report the performance of FISH and FSH for spaced seed Q7 in details over all datasets.

The results are in line with the above observations and FISH has better speed up across all datasets. However, for FISH the improvement on long reads datasets is substantial with respect to FSH.

### Multiple spaced seed hashing

Several tools exploit the power of spaced seeds by using a combination of such patterns, in order to further improve their performances in terms of quality. Therefore, the simultaneous computation of the hashing of several spaced seeds at once can come very useful in such contexts.

**Fig. 5:**
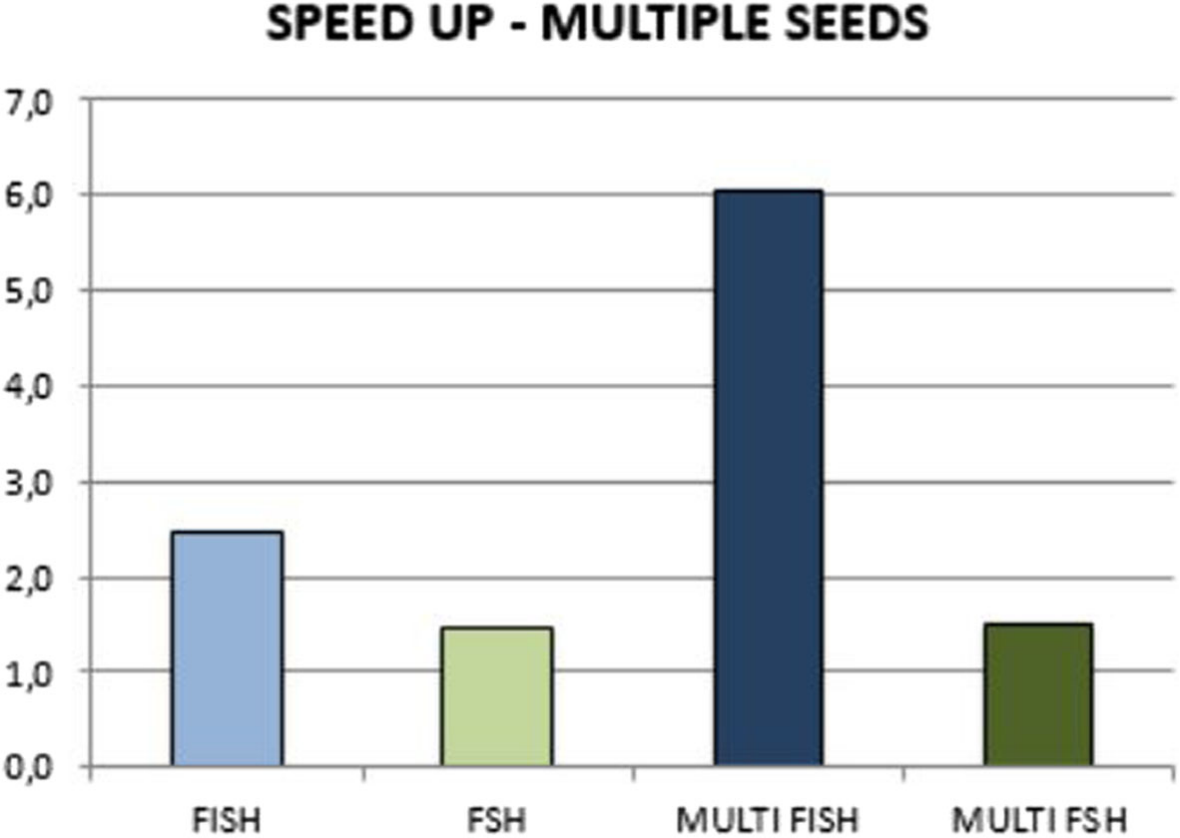
Speedup of FSH and FISH with the multiple spaced seeds hashing (dark green and dark blu) and with each spaced seed hashed independently (light green and light blu)

Figure 5 reports the speed up of FISH and FSH when computing the hash of spaced seed independently (light blu and light green), and simultaneously as multiple spaced seeds (dark blu and dark green).

**Fig. 6:**
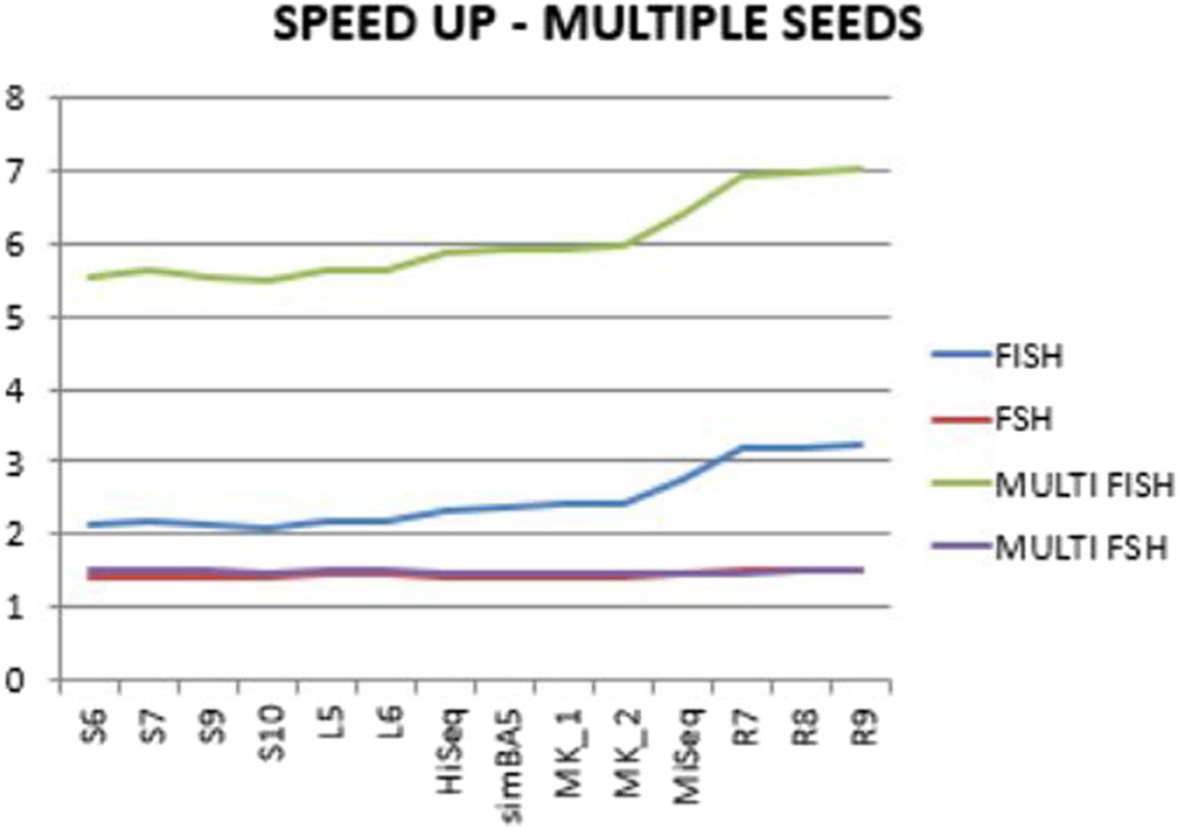
Details of the time speedup of FISH and FSH for the multiple spaced seeds hashing on different datasets

The use of multiple spaced seeds simultaneously increases the speed up of both methods. However, FSH improves from 1.45 to 1.49 whereas FISH from 2.48 to 6.03. On this experiment the advantage of FISH is gain substantial, where it can hash multiple spaced seeds 4 times faster than FSH. A detailed analysis of the performance on different datasets can be found in Fig. 6. Similarly to Fig. 3 we can observe that the speed up increases on long reads datasets.

### The impact of reads length and spaced seeds weight

These experiments aim at posing in evidence the impact on the speed up of reads length and spaced seeds density. We generated with rasbhari [22] different sets of nine spaced seeds with lengths from 16 to 45 and weights in the range from 11 to 32, see the Additional file 1: Tables S1-S5.

**Fig. 7:**
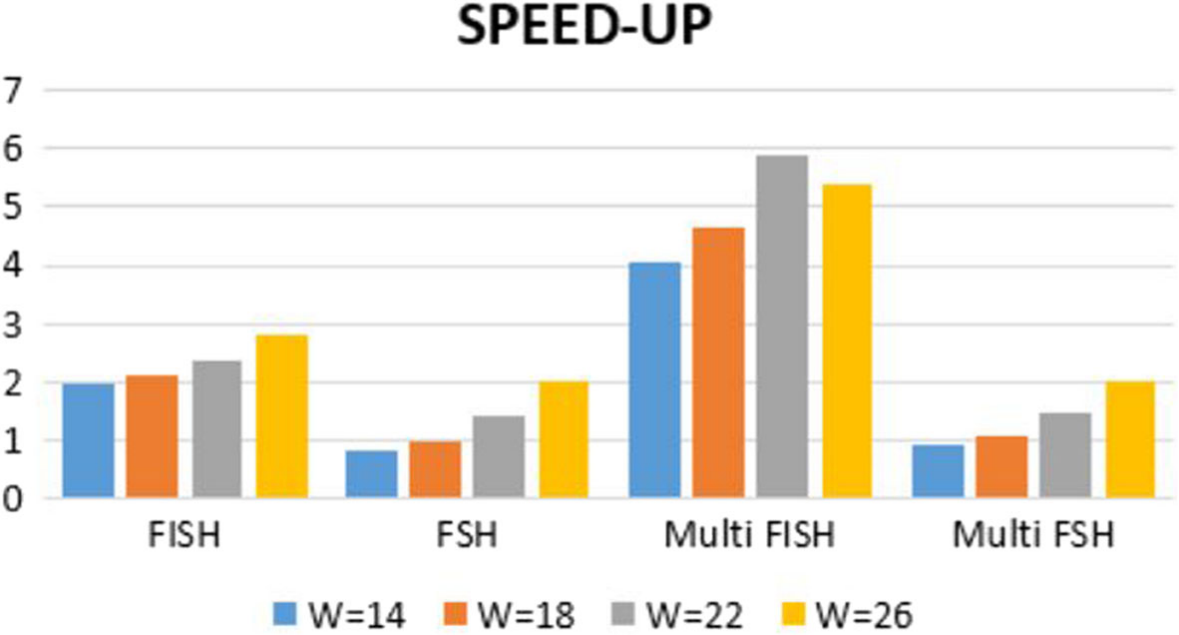
The speedup of FISH and FSH as a function of the spaced seeds density (*L*=31 and W varies)

In Fig. 7 we compare the speedup of FISH and FSH on spaced seeds with the same length *L*=31, while varying the weight *W*. It can be observed that the speed up of both FISH and FSH increases as the weight *W* increases. A possible explanation is the following. If a spaced seed has an higher weight, then the ability of FISH to use the partial hashes computed in the k-mers tables increases, and this will results in a better speed up. This behavior is consistent for both FISH and FSH, with the only exception of the speedup of FISH on multiple spaced seeds with *W* =22 and *L* =31. These are the seeds used in the first experiments and reported in Table 2. As opposed to the other set of seeds that have been created all with same tool and minimizing overlap complexity, these seeds have been created with different methods and thus they might expose more overlaps, allowing for a better speedup. On the other hand if the density *W*/*L* of spaced seeds weight with respect to the length is low, than both FISH and FSH will have poor performance. For example, if *W*/*L* is below 0.3 than the standard hashing computation is in general faster. On extreme cases, like the spaced seeds reported in [40], with *W*=12 and *L*=112 FISH and FSH might not be of help.

**Fig. 8:**
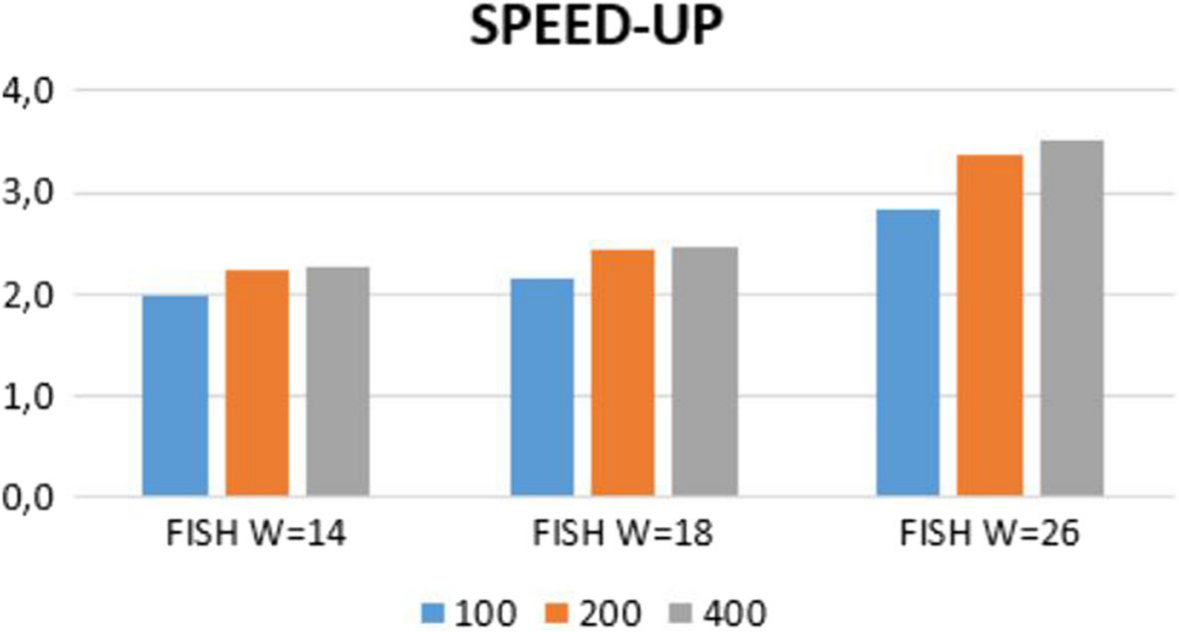
The speedup of our approach with respect to the standard hashing computation as a function of reads length (100, 200, 400) and the spaced seeds weight *W* (all with the same density)

In Fig. 8 we compare the speedup of FISH while varying the reads length, as a function of spaced seeds density (fixed lenght *L*=31). We can note that the speedup grows with the reads length, a behavior observed also in Figs. 3 and 4.

## Discussion

In this paper, we address the problem of hashing genomic sequences through the lens of spaced seeds. Spaced seeds are widely used in many tasks related to sequence alignment and comparison. In fact, on the problem of sequence similarity detection spaced seeds have shown better performance than contiguous matches [19]. While the hashing of contiguous matches can be efficiently performed, for spaced seed this was not the case.

We have already propose a method, called FSH [35], to address this problem, but in this paper we introduce a new tool, FISH, based on different strategies. FSH is based on spaced seed auto-correlation and dynamic programming, while FISH builds an index of partial common hashings that can be reused multiple times.

In the results section, we have shown that FISH can improve substantially the performance in terms of speed up w.r.t. to FSH and the traditional hashing of spaced seeds. This advantage is demonstrated on a number of different settings, varying spaced seeds density and reads length.

The speed up of FISH increases as the length of the reads grows. This is a desirable property if we consider that modern sequencing technologies can produce longer reads. Also, if spaced seeds with high density are required, FISH indexing strategy outperforms the other methods. One interesting direction of investigation is the use of long and sparse spaced seed, i.e. with very low density, for which FISH and FSH are not suited. It remains an open problem if an alternative hashing method can further improve the hashing computation, closing the gap with the fast hashing of k-mers.

## Conclusions

In this study we presented FISH, an indexing-based approach for speeding up the computation of rolling hash for spaced seeds. In our experiments FISH was able to compute the hashing values of spaced seeds with a speedup, on average and with respect to the traditional approach, between 1.9× (single) to 6.03× (multi), depending on the structure of the spaced seeds and on the reads length.

## Additional file

Additional file 1 Supplementary Tables. (PDF 45.9 kb)

## Abbreviations

NGS: Next generation sequencing
FSH: Fast spaced seed hashing
FISH: Fast indexing for spaced seed hashing
