# Supplementary material for: Efficient computation of spaced seed hashing with block indexing
Source: BMC Bioinformatics. 2018 Nov 30;19(Suppl 15):441. doi: 10.1186/s12859-018-2415-8 (PMC6266934; doi:10.1186/s12859-018-2415-8)
Supplement: Supplementary file 1 — Supplementary Tables. (PDF 45.9 kb) [file 12859_2018_2415_MOESM1_ESM.pdf]

Additional File 1

Table 1 Nine spaced seeds with $W = 11$ and length 16 computed with rasbhari minimizing overlap complexity.

Q10	1011101100101110
Q11	1100111100011110
Q12	1101011100011110
Q13	1101101110111000
Q14	1110110010110110
Q15	1111001100101110
Q16	1111001101110010
Q17	1111100011010110
Q18	1111110001011100

Table 2 Nine spaced seeds with $W = 32$ and length 45 computed with rasbhari minimizing overlap complexity.

Q19	100111111111111001001011110111100111011011011 1
Q20	101001111001011111011110111111110001010111111
Q21	110100110101111100011111011011111111111110001
Q22	110101011001100111110101110011001111111111111
Q23	110111011111111110110111110101001000001111111
Q24	111011100111010001101111001111110011111110111
Q25	111100011011010010011111111011111111100011111
Q26	111101001101110011101110101011101110111011111
Q27	111101101111110001111111000101110101110111011

Table 3 Nine spaced seeds with $W = 26$ and length 31 computed with rasbhari minimizing overlap complexity.

Q19	1111101110111011111101101111111
Q20	1111111011111111111111010101101
Q21	1110111011011111111110011111111
Q22	1111111111100111101111111110011
Q23	1111011111111111101011011110111
Q24	1101101101111100111111111111111
Q25	1011110101111101111111111111101
Q26	1111101111110111111110111110011
Q27	1011111111110101111101011111111

Table 4 Nine spaced seeds with $W = 18$ and length 31 computed with rasbhari minimizing overlap complexity.

Q19	1011010110010101111011010110001
Q20	1011001001011011111101010101001
Q21	1010101001011101100110000111111
Q22	1110110011000010001111011110011
Q23	1010011111101111101011000000011
Q24	1100100101010000110101111111011
Q25	1011100100110101111100010111001
Q26	1110101011100111101010011100001
Q27	1011011110001011101000100011111

Table 5 Nine spaced seeds with $W = 14$ and length 31 computed with rasbhari minimizing overlap complexity.

Q19	1110000100110100110110001010001
Q20	1011000001001011111101000100001
Q21	1010000000100110110011000011011 1
Q22	1110010001000010000111011100011
Q23	1010000111100111100011000000011
Q24	1000100000001000001010111111011
Q25	1011100000100101011100010110001
Q26	1000101011100101101010001100001
Q27	1011010110001010101000000010111
